# Camel Milk Beneficial Effects on Treating Gentamicin Induced Alterations in Rats

**DOI:** 10.1155/2014/917608

**Published:** 2014-12-03

**Authors:** Abdulrahman K. Al-Asmari, R. Abbasmanthiri, Abdulrahman M. Al-Elewi, Saud Al-Omani, Saeed Al-Asmary, Sarah A. Al-Asmari

**Affiliations:** ^1^Department of Research Center, Prince Sultan Military Medical City, P.O. Box 7897 (775-S), Riyadh 11159, Saudi Arabia; ^2^Department of Urology, Prince Sultan Military Medical City, P.O. Box 7897, Riyadh 11159, Saudi Arabia; ^3^Department of Surgery, Prince Sultan Military Medical City, P.O. Box 7897, Riyadh 11159, Saudi Arabia; ^4^Department of F & C Medicine, Prince Sultan Military Medical City, P.O. Box 7897, Riyadh 11159, Saudi Arabia; ^5^Department of Dentistry, Prince Sultan Military Medical City, P.O. Box 7897, Riyadh 11159, Saudi Arabia

## Abstract

The potential effect of camel milk (CM) against gentamicin (GM) induced biochemical changes in the rat serum was evaluated. Four groups of six albino rats were used for control, CM fed, injected with GM(i.p.), and then fed and injected with GM. The results showed that the administration of GM significantly altered the levels of aspartate aminotransferase (AST), alanine aminotransferase (ALT), alkaline phosphatase (ALP), and lactate dehydrogenase (LDH) activity in rat serum. CM restored these parameters to almost their normal range in group IV. Additionally, the present study showed that injection of rats with gentamicin caused an increase in malondialdehyde (MDA) and myeloperoxidase (MPO) activity while the antioxidant enzymes like superoxide dismutase (SOD) and glutathione s-transferase (GST) activity decreased significantly (*P* ≤ 0.05). Administration of CM significantly (*P* ≤ 0.05) inhibited the formation of MDA and activity of MPO and upregulated the antioxidant enzymes (SOD and GST) activity. The overall findings of this study demonstrated that pretreatment with CM gave protection against GM induced hepatic damage possibly by inhibiting oxidative stress and inflammation, and hence camel milk can be identified as a new therapeutic agent.

## 1. Introduction

The unique characteristics of CM are seen in it is often used to counter diseases such as diabetes and hepatic and microbial infections [1–3], in addition to the reported improvement effects in blood and renal and hepatic functions [4]. Low in cholesterol, sugar, and protein but having higher levels of minerals as electrolytes, vitamins, and insulin, CM is presented as unique compared to the milk of other ruminant mammals [5]. Some active CM proteins were successfully studied, with specific antibacterial and antiviral promising results [1, 6, 7]. Insulin dependent diabetes patients could reduce their insulin dose by one third when taking raw CM [8–10], which refers to some control on metabolic and autoimmune diseases. In this field, autoimmune diseases, diabetes, and respiratory and various types of tuberculosis were extensively studied [11], in addition to specific hepatic intoxication studies [12–17].

Aminoglycoside group of antibiotics, which include gentamicin (GM), have beneficial treatment effects against most of the life-threatening Gram-negative microorganisms [18], though their side effects encompass considerable nephrotoxic and hepatotoxic complications that were contracted by almost one third of the treated patients [19], especially in the GM nephrotoxic alterations field [20, 21]. In the present study, the major objectives were to evaluate the effect of CM consumption on GM induced biochemical changes in experimental rats by measuring the serum activity and levels of lipid peroxidation (MDA), MPO, GST, SOD, AST, ALT, ALP, and LDH.

## 2. Materials and Methods

### 2.1. Camel Milk

Camel milk was collected from local suburbs by hand milking. The samples were collected in sterile screw bottles and kept in cool boxes until transported to the laboratory. The rats were given this fresh milk (5 mL/rat/day) as such without any further treatment.

### 2.2. Animals

A total of 24 male albino rats (200–250 g) were obtained from animal house facility in the Research Center, Prince Sultan Military Medical City (PSMMC), Riyadh, Saudi Arabia. Rats were acclimated for ten days before starting the experiment. All animals were housed in standard cages (six rats/cage), feeding with standard laboratory diet and water* ad libitum*. The experimental animals were housed in air-conditioned rooms at 21–23°C and 60–65% of relative humidity and kept on a 12 h light/12 h dark cycle. The animals received humane care in accordance with the Guide for the Care and Use of Laboratory Animals, published by Ethics of Scientific Research Committee of PSMMC.

### 2.3. Gentamicin

80 mg (2 mL vials) gentamicin was obtained from Parkin Remedies.

### 2.4. Experimental Design

Animals were divided into four groups of six rats each. Group I served as control and received only normal saline injections (0.2 mL, i.p.). Group II was given CM only (5 mL/rat/day for fifteen days, orally). Group III was injected with GM (2 mL vials) only (80 mg/kg b.wt for the last ten days, i.p.). Group IV was given CM (alone for first five days) and then injected with GM (for 10 days).

### 2.5. Collection of Blood Serum

At the end of the experiment, that is, on day sixteen, the overnight fasted animals (the control and experimental animals) were sacrificed under mild ether anesthesia. Blood samples were collected by cardiac puncture before incision of the abdomen; 5 mL of blood samples was collected in plain tubes and serum was separated and frozen at −80°C until the time of analysis.

### 2.6. Biochemical Analysis

Commercial diagnostic kits (United Diagnostic Industry, UDI, Dammam, Saudi Arabia) were used for determination of ALT, AST, ALP, and LDH. Concentration of the biochemical constituents was calculated according to the manufacturer instructions.

### 2.7. Estimation of MDA

The concentration of thiobarbituric acid reactive substances (TBARS) was determined by the method of Ohkawa et al. [22]. In brief, the reaction mixture contained 0.1 mL of serum, 0.2 mL of sodium dodecylsulfate, 1.5 mL of acetic acid, and 1.5 mL of aqueous solution of TBA. The pH of 20% acetic acid was preadjusted with 1 M NaOH to 3.5. The mixture was made up to 4 mL with distilled water and heated at 95°C for 1 h, in a water bath. After cooling, 1 mL of distilled water and 5 mL of mixture of n-butanol and pyridine (15 : 1) were added and mixture was shaken vigorously on a vortex mixer. The absorbance of the upper organic layer was read at 532 nm using UV-VIS spectrophotometer.

### 2.8. Determination of Glutathione S-Transferase (GST) Activity

The method of Habig et al. [23] was used with some modifications to estimate the activity of glutathione s-transferase (GST). In a final volume of 2 mL, the reaction mixture consisted of 0.1 mole phosphate buffer, 1 mM reduced glutathione, 1 mM 1-chloro-2,4-dinitrobenzene (CDNB), and serum. The GST activity determined as nM CDNB conjugate formed min/ml using a molar extinction coefficient of 9.6 × 10^3^ M^−1 ^cm^−1^.

### 2.9. Determination of Superoxide Dismutase (SOD) Activity

Superoxide dismutase (SOD) activity was determined according to the method described by S. Marklund and G. Marklund [24]. The reaction mixture consisted of 0.5 mL of tris-buffer (50 mM; pH-8.2), 0.5 mL pyrogallol (0.5 mM), and 0.5 mL EDTA (1 mM), in different volumes, 0.025 mL, 0.05 mL, 0.075 mL, and 0.1 mL of serum. The change in absorbance was recorded at 420 nm. Activity was reported by its ability to inhibit 50% reduction of pyrogallol and the result is expressed as U/mL.

### 2.10. Determination of Myeloperoxidase (MPO)

The activity of the inflammatory marker myeloperoxidase (MPO) in the serum was measured with some modification according to the method of Barone et al. [25]. MPO in the serum was assayed by mixing 0.1 mL of serum with 2.9 mL of 50 mM potassium phosphate buffer (pH 6.0) containing 0.167 mg/mL o-dianisidinedihydrochloride (Sigma) and 0.0005% hydrogen peroxide (ICN Pharmaceuticals, Irvine, CA). The change in absorbance at 460 nm was measured for 3 min by using a UV-visible spectrophotometer (UV-160A, Shimadzu, Japan). MPO is expressed in units of activity per mL of serum, with 1 unit being the quantity of enzyme able to convert 1 *μ*mol of hydrogen peroxide to water in 1 min at room temperature.

### 2.11. Statistical Analysis

Results were expressed as means ± standard error of mean (SEM). The significance of differences was calculated by SPSS program (version 20) using Student's *t*-test; *P* ≤ 0.05 was considered statistically significant.

## 3. Results

### 3.1. Effect of Camel Milk and Gentamicin on Biochemical Enzyme Levels

The activity of ALT, AST, ALP, and LDH was estimated in serum samples as the liver function biomarkers. These results are given in Table 1. The GM treatment markedly affected the liver specific enzymes. A significant increase was found in serums ALT, AST, ALP, and LDH activity in GM treated group as compared to control group (29.15 ± 1.85 to 39.70 ± 2.17 (*P* < 0.001); 77.08 ± 2.5 to 128.63 ± 6.31 (*P* < 0.001); 58.80 ± 6.90 to 123.29 ± 9.09 (*P* < 0.01); 332.89 ± 17.59 to 466.93 ± 15.54 (*P* < 0.01), resp.). This result suggests that these hepatic biomarkers were elevated in the serum due to release of the enzymes from damaged liver. However, a significant decrease was observed in the respective serum activity of above mentioned biomarkers of rats in group IV as compared to group III (39.70 ± 2.17 to 33.62 ± 3.0; 128.63 ± 6.31 to 111.02 ± 5.25 (*P* < 0.05); 123.29 ± 9.09 to 87.29 ± 5.96 (*P* < 0.05); 466.93 ± 15.54 to 398.81 ± 15.76 (*P* < 0.05)).

### 3.2. Effect of Camel Milk and Gentamicin on Lipid Peroxidation

**Figure 1:**
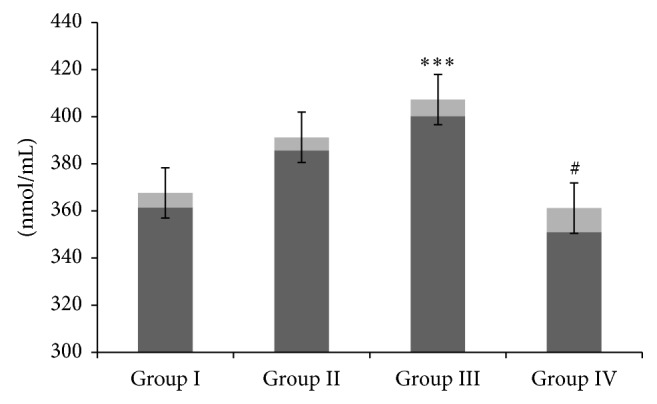
Effect of camel milk and gentamicin on lipid peroxidation. Effect of CM administration on GM induced renal lipid peroxidation. Values are expressed as mean ± SEM (*n* = 6). ^***^ *P* < 0.001 shows significant difference in group III when compared with group I. ^#^ *P* < 0.05 shows significant difference in group IV when compared with group III.

Camel milk inhibited lipid peroxidation caused by GM administration in terms of MDA, a well-known biomarker of oxidative stress. Administration of GM led to a significant elevation in the level of MDA in group III compared to controls (*P* < 0.001). Administration with CM in group IV was significantly (*P* < 0.05) effective in amelioration of MDA formation as compared to group III. There was no significant change observed in the level of MDA between controls and CM-treated animals (Figure 1).

### 3.3. Effect of Camel Milk and Gentamicin on Antioxidant Enzymes

**Figure 2:**
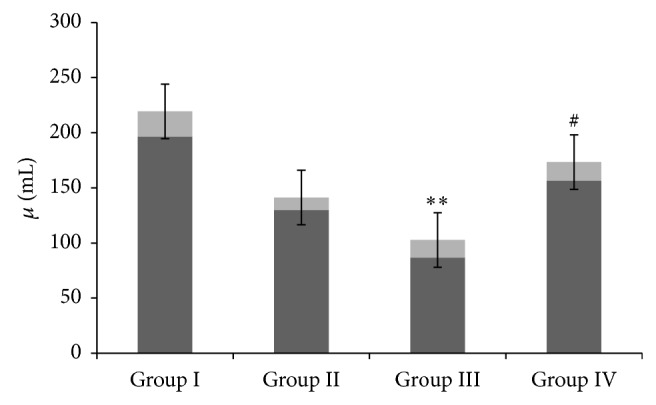
Effect of camel milk and gentamicin on SOD enzymes. Effect of CM intake and GM on renal SOD activity. Values are expressed as mean ± SEM (*n* = 6). ^**^ *P* < 0.01 shows significant difference in group III when compared with group I. ^#^ *P* < 0.01 shows significant difference in group IV when compared with group III.

**Figure 3:**
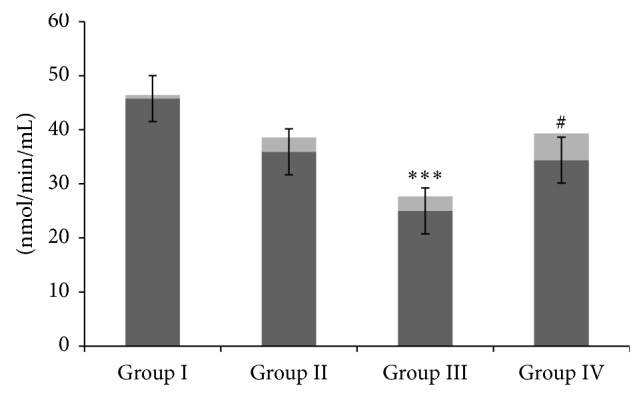
Effect of camel milk and gentamicin on GST enzymes. Effect of CM treatment on GM induced renal GST activity. Values are expressed as mean ± SEM (*n* = 6). ^***^ *P* < 0.001 shows significant difference in group III when compared with group I. ^#^ *P* < 0.05 shows significant difference in group IV when compared with group III.

The effect of CM administration on GM induced depletion in the activity of SOD and GST enzymes was examined and the results were shown in Figures 2 and 3, respectively. We have observed that there was a significant (*P* < 0.01,  *P* < 0.001) depletion in the activity of these antioxidant enzymes in group III as compared to controls. However, CM administration in group IV significantly restored the activity of antioxidant enzymes when compared with group III (*P* < 0.01, *P* < 0.05). There was no significant difference observed between groups I and II (Figures 2 and 3).

### 3.4. Effect of Camel Milk and Gentamicin on Myeloperoxidase Activity

**Figure 4:**
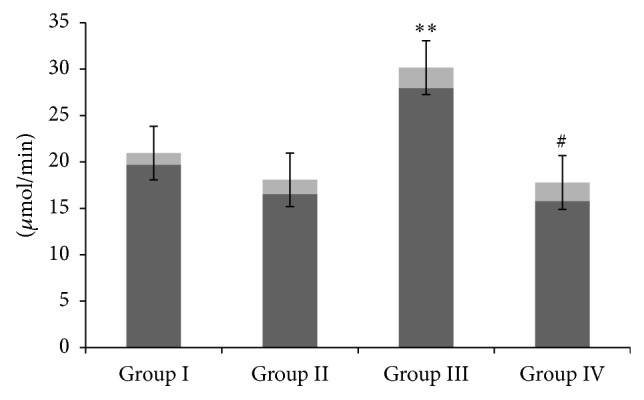
Effect of camel milk and gentamicin on myeloperoxidase activity. Effect of CM administration on GM induced renal MPO activity. Values are expressed as mean ± SEM (*n* = 6). ^**^ *P* < 0.01 shows significant difference in group III when compared with group I. ^#^ *P* < 0.05 shows significant difference in group IV when compared with group III.

We have observed that there was a significant increase in the activity of MPO in only GM treated group III as compared to group I (*P* < 0.01). However, CM administration in group IV significantly restored the activity of MPO when compared with group III (*P* < 0.05). There was no significant difference observed between groups I and II (Figure 4).

## 4. Discussion

The serum hepatic biomarkers, AST and ALT, activity were increased significantly in rats injected with GM in group III as compared to controls (group I), which suggest the release of enzymes of the damaged hepatocytes. These cytoplasmic enzymes released in the circulation were suggested to be acting on the signal transduction pathways leading to membrane cellular permeability, according to previous reports [26–28]. GM toxicity enhances oxidative stress and the so formed reactive oxygen species (ROS) that exhaust the countering antioxidant enzymes and biomolecules [27–30], referring to further impaired pathway processes [31]. The significant increase in the LDH of GM injected rats compared to controls is also in agreement with previously published findings [3, 13]. It was seen that the MDA (lipid peroxidation marker) and inflammatory marker MPO activity were significantly increased, whilst the endogenous antioxidants GST and SOD were decreased. This finding is consistent with researchers' reports that attribute the inactivation of enzymes to their cross-linking with MDA that lead to increased accumulation of superoxide free radicals inducing more lipid peroxidation [13, 16, 28, 30, 32, 33].

On the other hand, treatment with camel milk was found to suppress the increase of serums AST and ALT activity induced by GM treatment in rats. This finding implies that CM has the potential to repair and protect liver tissues to be affected by GM injection, through membrane stabilizing and leakage prevention of intracellular enzymes. It could be interpreted that the repair and healing process of hepatic parenchymal cells should lead to reversal of serum transaminase levels [34]. CM protective effects had been reported previously in some related topics [3, 17, 35, 36], which attribute the harmful hepatic effects restoration, back to normal physiology, to CM consumption that affects the regeneration or protection of hepatocyte membrane integrity [37].

The serum antioxidant system enzymes (GST and SOD) and inflammatory marker (MPO) were also restored to normal optimum levels with CM consumption, which was attributed to its high contents of antioxidant vitamins, minerals, other elements [30], several potential therapeutic effects [8, 9], and disease resistance [2, 38–42]. Dysfunctional mitochondria are responsible for the production of excessive superoxides, which are substrates for the conversion to inflammatory biomarkers [43] that mediate for contraction of some diseases [44–46].

Some minerals and vitamin availability (as the case of abundant CM) enhance SOD levels and, oppositely, lower levels would lead to depletion [47] and it was shown in this study that SOD levels were significantly increased after CM administration. The reports of Barbagallo et al. [48] and Virginia et al. [49] have stressed the indispensable links between glutathione and other antioxidants with minerals and vitamins.

GM induced complications had been worked on with antioxidants by several researchers [21, 50, 51]. In Islamic communities, CM health benefits are popular, as has been stated by the Prophet Mohammed (PBUH) more than 1400 years ago. Several previous studies had experimentally proved the beneficial effects of camel milk. Some components in cow milk that are responsible for allergies are not found in camel milk whose protein components (beta-casein) are different [52] and decisive in curing and preventing food allergies [53]. Hence, it is used as a therapeutic agent in several diseases and also as antimicrobial [54–57] and antiviral remedy [57, 58]. In addition to that camel milk contains a number of compatible immunoglobulins compared with human ones [59]. With respect to insulin, camel milk contains higher levels than the contents of cow and buffalo milk [60]. Recent studies have reported CM as possessing several beneficial characteristics [9, 17, 61, 62] but still the exact mechanisms and active remediation effects of CM against GM induced tissue damage are not fully investigated.

## 5. Conclusion

The present findings show that administration of CM exerts significant hepatoprotective and nephroprotective effects in gentamicin-treated rats. Further investigations are required to explore exactly the mechanisms of action of CM against gentamicin-induced physiological changes. Finally, the present study identifies new areas of research for development of better therapeutic agents for liver, kidney dysfunction, and other diseases.

## Figures and Tables

**Table 1:** Effect of camel milk on the activity of alanine aminotransferase (ALT), aspartate aminotransferase (AST), alkaline phosphate (ALP), and lactate dehydrogenase (LDH).

Parameters	Group I (control)	Group II (CM)	Group III (GM)	Group IV (CM + GM)
ALT (U/L)	29.15 ± 1.85	31.65 ± 0.90	39.70 ± 2.17^a^	33.62 ± 3.0^b^
AST (U/L)	77.08 ± 2.5	68.37 ± 3.53^d^	128.63 ± 6.31^a^	111.02 ± 5.25^a,b,c^
ALP (U/L)	58.80 ± 6.90	77.07 ± 10.75^d^	123.29 ± 9.09^a^	87.29 ± 5.96^a,b,c^
LDH (U/L)	332.89 ± 17.59	212.04 ± 17.80^d^	466.93 ± 15.54^a^	398.81 ± 15.76^a,b,c^

Values are given as means ± SEM for groups of six animals each. Values are statistically significant between two groups *P* ≤ 0.05. ^a^Control group compared with gentamicin group; ^b^control groups compared with camel milk and gentamicin group; ^c^gentamicin groups compared with camel milk and gentamicin group; ^d^control groups compared with camel milk group.
